# Analysis of Cervix Intraepithelial Neoplasia Prevalence in the Kurdistan Region, Iraq

**DOI:** 10.7759/cureus.45725

**Published:** 2023-09-21

**Authors:** Fatima K Khalid, Mahabad S Ali

**Affiliations:** 1 Obstetrics and Gynecology, College of Medicine, University of Zakho, Zakho, IRQ; 2 Internal Medicine, College of Medicine, Erbil Maternity Teaching Hospital, Erbil, IRQ

**Keywords:** vaginal problem, kurdistan, cervical intraepithelial neoplasia, pap smear, cervical cancer

## Abstract

Background: Cervix intraepithelial neoplasia (CIN) refers to abnormal changes in the squamous cells of the cervix, with more significant changes known as high-grade squamous intraepithelial lesions (HSIL) occurring in grades II and III.

Aim: The study aimed to estimate the prevalence of cervical intraepithelial neoplasia and explore potential risk factors for HSIL among women living in the Kurdistan region, Iraq.

Materials and Methods: The research encompassed a sample size of 1300 female participants whose Papanicolaou (pap) smears were taken in several hospitals located within the Kurdistan region. The objective of the study was to determine the prevalence of cervical infection among these women. This was a multi-centre study conducted from January 2021 to the end of December 2021 for the determination of pap smears and the relationship between CIN and cervical cancer. Only 120 (9.23%) of those 1300 participants suffered from cervical or vaginal problems. A pap smear, also known as a pap test, is a routine screening procedure used to detect abnormal cervical cells that may indicate cervical cancer or precancerous conditions. The procedure involves collecting a sample of cells from the cervix.

Results: The most important details in this study are the age groups and percentages of women who have undergone a pap smear. There were four women who tested positive for cervical cancer, accounting for 3.33% of the 120 participants with cervical or vaginal problems. However, there were 116 (96.67%) women who tested negative. With regard to the distribution of inflammation rates among the participants with cervical or vaginal problems, there were 114 (95%) women who suffered inflammation, whereas there were six (5%) women without inflammation. In each age group, the numbers of women with and without cervical cancer, as well as the corresponding percentages, were considered. The data suggest that the incidence of cervical cancer tends to increase with age since higher percentages were observed in older age groups. The study highlights the importance of regular screenings and age-specific cervical cancer prevention and detection strategies to ensure early diagnosis and effective medical interventions.

Conclusion: The prevalence of cervical cancer cases was relatively low, with only 0.31% of the total participants diagnosed with cervical cancer. The majority, accounting for 99.69%, did not have cervical cancer. These results highlight the importance of pap smear screenings as a valuable tool for early detection and prevention of cervical cancer. They also highlight the importance of regular screenings, especially for younger women, to detect and treat cervical abnormalities at an early stage.

## Introduction

Cervix cancer constitutes the second-most widespread cancer and remains the foremost cause of cancer-related fatalities among women across the globe, with an estimated 90% of deaths occurring in low and middle-income countries. Disparities in prevalence are stark, as regions like Sub-Saharan Africa and parts of Asia experience particularly high incidence rates. These disparities are compounded by limited access to healthcare and resources, resulting in a disproportionate burden. In these regions, the efficacy of Papanicolaou (Pap) smears, a cornerstone of cervical cancer screening, is highlighted. However, challenges persist in achieving optimal screening rates, with some areas reporting Pap smear positivity rates as low as 20%, underscoring the need for enhanced awareness, education, and healthcare infrastructure to combat this preventable disease and bridge the gap in cervical cancer care [1, 2].

Cervix intraepithelial neoplasia (CIN) refers to abnormal changes in the squamous cells of the cervix, with more significant changes known as high-grade squamous intraepithelial lesions (HSIL) occurring in grades II and III. If left untreated, at least 25% of HSIL will progress to carcinoma in situ or even invasive cancer [3]. Thus, identifying risk factors for HSIL is crucial for early detection and treatment. Research suggests that the progression of CIN is influenced by human papillomavirus (HPV) infection and certain lifestyle factors. HPV is a key player in the development of cervical cancer and precursor lesions, particularly subtypes 16 and 18 [4-6]. Previous studies have detected dose-response relationships between smoking intensity, duration, and cervix cancer [5]. However, smoking does not show a significant association with adenocarcinoma of the cervix, which typically accounts for less than 10% of all cervical cancers [6]. In recent years, extensive evidence from epidemiological and experimental studies has highlighted the role of inflammation in the development of CIN and cervical cancer [7]. Indeed, anti-inflammatory drugs have been shown to reduce the risk of cancer and precancerous lesions in epidemiological studies [8]. Factors related to sexual behaviour have also been associated with cervical cancer and its precursors. Bacterial vaginosis and Trichomonas vaginalis infections have been significantly linked to persistent HPV infection and the development of cervical cancer [9]. Multiple sexual partners have also been shown as a factor in increasing the risk of HPV acquisition and cervical cancer [10]. The present study aims to estimate the prevalence of cervical neoplasia and explore potential risk factors for HSIL among women living in the Kurdistan region, Iraq.

## Materials and methods

The study, conducted from January 2021 to December 2021, included 1300 women who underwent Pap smears for the conclusion of cervical infection in hospitals throughout the Kurdistan region. Only 120 of the 1300 participants suffered from cervical or vaginal problems. Ethical approval was obtained from the University of Zakho with the institutional review board number IEC/2E/2019/32. A Pap smear, also known as a Pap test, is a routine screening procedure used to detect abnormal cervical cells that may indicate cervical cancer or precancerous by collecting a sample of cells from the cervix. The sample was processed in the same way in all instances.

Sample preparation: The collected cells were then placed on a glass slide or in a liquid medium to preserve them for laboratory analysis.

Speculum removal: Once the sample was obtained, the speculum was carefully removed from the vagina.

Follow-up care: The participants were instructed to get dressed after the sample collection and to discuss any concerns or questions with their healthcare provider. The sample was given to a lab for examination, and the results were communicated to them.

It's important to note that the Pap smear procedure is generally quick and straightforward, typically lasting only a few minutes. It is recommended for women to have regular Pap smears as part of their preventive healthcare routine as the test can help detect early signs of cervical abnormalities or cancer, which is when treatment is most effective.

## Results

The current study included 1300 women who underwent Pap smears for the conclusion of cervical infection in several parts and hospitals throughout the Kurdistan region. Only 120 of the 1300 participants suffered from cervical or vaginal problems. Further, only four (0.31%) of the 1300 participants developed cervical cancer (Table 1).

**Table 1:** Prevalence of cervical cancer among the participants who underwent Pap smears

Presence of cervical cancer	Number	Percentage (%)
Present	4	0.31
Absent	1296	99.69
Total	1300	100

The following table details the distribution and percentages of patients with cervical or vaginal problems in each age group (Table 2). Out of the 120 patients with cervical or vaginal problem, 17 (14.17%) were in the age group of 17-30 years. There were 38 (31.67%) patients in the 31-39 year age group, 39 (32.50%) patients in the 40-49 year age group, 21 (17.50%) in the 50-59 year age group, and only five (4.17%) in the 60-69 year age group. 

**Table 2:** Distribution of participants with cervical or vaginal problems by age

Age groups (years)	Number	Percentage (%)
17-30	17	14.17
31-39	38	31.67
40-49	39	32.50
50-59	21	17.50
60-69	5	4.17
Total	120	100

Table 3 shows the distribution of inflammation rates among participants with cervical or vaginal problems. Inflammation affected 114 (95%) of the participants with cervical or vaginal problems; the other six (5%) patients did not experience inflammation.

**Table 3:** Inflammation rates among participants with cervical or vaginal problem

Inflammation	Number	Percentage (%)
Absent	6	5.00
Present	114	95.00
Total	120	100

Table 4 presents the prevalence and percentages of cervical cancer in participants with cervical or vaginal problems in each age group.

**Table 4:** Presence or absence of cervical cancer in participants with cervical or vaginal problems by age group

Age groups (years)	Cervical cancer	
	Absent	Present
17-30	17	0
	100%	0%
31-39	38	0
	100%	0%
40-49	37	2
	94.87%	5.13%
50-59	20	1
	95.24%	4.76%
60-69	4	1
	80%	20%
Total	116	4
	96.67%	3.33%

Table 4 provides insights into the relationship between the age groups of women with cervical or vaginal problems and the presence of cervical cancer. In the age groups of 17-30 years and 31-39 years, no cases of cervical cancer were detected, resulting in a 0% incidence rate. However, among the 39 women aged 40-49 years, two individuals were diagnosed with cervical cancer, representing a 5.13% incidence rate. In the age group of 50-59 years (21 patients), one woman had cervical cancer, resulting in a 4.76% incidence rate. Similarly, for the age group of 60-69 years, one woman was diagnosed with cervical cancer; however, since there were only five patients within the age group, a higher incidence rate of 20% was reached.

## Discussion

The highest rate of women who underwent a pap smear and were diagnosed with cervical infections occurred within the age group of 40-49 years. This finding implies that a significant number of women within this age range were affected by cervical infections, as detected through the Pap smear screening method. This may be because the age range of 40-49 years is a period when women are typically still in their reproductive years and may be sexually active. Cervical infections, particularly those caused by the human papillomavirus (HPV), are often associated with sexual activity. HPV is a common sexually transmitted infection and is a significant risk factor for developing cervical cancer [7, 8].

A Pap smear, also known as a Pap test, is a screening procedure used to detect abnormal changes in the cells of the cervix. These changes can indicate the presence of precancerous or cancerous conditions. Regular Pap smear screenings are recommended for women as a preventive measure for early detection of cervical abnormalities and potential signs of cancer. Pap smears involve collecting cells from the cervix to examine for any abnormal changes that may indicate an infection or pre-cancerous or cancerous conditions. By detecting infections and abnormalities early, appropriate medical interventions can be implemented to prevent the progression of cervical cancer [9, 10]. While this study highlights a higher incidence of cervical infection among women aged 40-49, it is important to note that cervical infections can occur at any age (see Table 2). Notably, within the age groups of 17-30 and 31-39, there was not one person from this age group with detected cancer, resulting in a 0% incidence rate for these age categories. HPV infections can resolve on their own without causing any significant health issues. However, persistent infections with high-risk strains of HPV can increase the risk of developing cervical cancer over time [11, 12]. It is therefore crucial for women in this age group to adhere to regular cervical cancer screenings, follow recommended guidelines for HPV vaccination, practice safe sex, and consult with healthcare professionals for personalized advice on preventive measures and appropriate management of cervical infections or abnormalities [13, 14]. The finding of the prevalence of cervical cancer at 0.31% among the female participants who took a Pap smear test is a significant observation. It highlights the importance of regular cervical cancer screening through Pap smears as a means of early detection and prevention, especially for women who are at higher risk due to factors such as age, sexual activity, or exposure to known risk factors like HPV infection.

The fact that 0.31% of the studied women who underwent a Pap smear were found to have cervical cancer suggests that the screening program was effective in identifying cases at an early stage [15, 16]. Early detection of cervical cancer is crucial because it allows for timely intervention and treatment, which can greatly improve the chances of successful outcomes. By identifying precancerous or cancerous cells early on, healthcare providers can initiate appropriate interventions such as further diagnostic tests, biopsies, or treatment options like surgery, radiation therapy, or chemotherapy [17]. The results serve as a reminder that even seemingly healthy individuals can be affected by cervical cancer and that routine screening can help in detecting and addressing the disease at an early stage [18]. It is worth noting that the prevalence rate may vary in different populations or regions due to variations in risk factors, access to healthcare, and awareness of the importance of regular screening. Nonetheless, the finding of a 0.31% prevalence rate among the studied women emphasizes the significance of Pap smears as a valuable tool in cervical cancer prevention and control efforts [19]. They provide evidence of the prevalence of cervical cancer among women, emphasizing the need for continued efforts to promote awareness, accessibility, and utilization of cervical cancer screening programs. By identifying and addressing cervical cancer early, we can make significant progress in reducing the burden of this disease and improving health outcomes [20].

The data provided also reveal the distribution of inflammation rates among the female participants. Out of the total number of women with cervical or vaginal problems, 95.0% were found to have developed inflammation, while only 5.0% showed no signs of inflammation. The high percentage of women with inflammation suggests that inflammation is a common occurrence among women with cervical or vaginal problems. Inflammation can be caused by various factors, such as infections, irritants, or immune responses. It is important to note that inflammation itself does not necessarily indicate a serious health condition as it can be a natural response of the body to protect and heal itself [21]. However, persistent or chronic inflammation may require further investigation and appropriate medical management [22]. On the other hand, the low percentage of women without inflammation indicates that this group represents a minority among those who underwent a Pap smear. These women may have a lower likelihood of inflammation in the cervix due to various factors such as a healthy immune system or the absence of underlying conditions that may trigger inflammation. Overall, the high rate of inflammation among women who underwent a Pap smear highlights the importance of considering inflammation as a potential factor in the evaluation of cervical health. Proper identification and management of inflammation, along with regular screenings, can help monitor and address any potential underlying conditions or concerns. It is essential for healthcare providers to interpret these findings in the context of each individual's medical history and symptoms to provide appropriate care and guidance [1].

The interpretation of Table 4 reveals a clear association between age groups and the presence or absence of cervical cancer among women with cervical or vaginal problems. In the younger age groups of 17-30 years and 31-39 years, no women were diagnosed with cervical cancer, resulting in a 0% incidence rate. This suggests that cervical cancer is quite rare in these age groups. As we move to the 40-49 age group, the incidence rate is 5.13%, showing a moderate risk of cervical cancer. In the age group of 50-59, the incidence rate is 4.76%, which is slightly lower but still represents a moderate risk. For women aged between 60 and 69 years, the incidence rate rises to 20%, suggesting that the risk of cervical cancer increases in this older age group.

This study has some limitations. For instance, a larger sample size would have provided more robust data and increased the generalisability of the findings. Further, the participants selected were women who attended specific parts and hospitals in the Kurdistan region of Iraq. This selection method may have introduced bias and limited the representativeness of the study population as it may not accurately reflect the global prevalence and risk factors of cervical neoplasia. Additionally, the accuracy and reliability of the collected data could be influenced by the participants' understanding of the questions, memory limitations, or reluctance to disclose certain information. Future studies with larger sample sizes, diverse populations, and longitudinal designs would provide a more comprehensive understanding of these factors.

## Conclusions

In summary, the study found a relatively low percentage (0.31%) of cervical cancer cases among women who underwent Pap smear screenings. The majority (99.69%) of the participants did not have cervical cancer. Additionally, inflammation rates among the patients with cervical or vaginal problems were high (95%), indicating the need for further investigation and appropriate management. The data also revealed a clear association between age and cervical cancer, with higher percentages observed in older age groups. These findings underscore the significance of age-specific cervical cancer prevention strategies and regular screenings to detect abnormalities at an early stage. Implementing effective screening programmes and raising awareness can significantly improve outcomes and reduce the impact of cervical cancer on women's health.
